# Knockdown of lncRNA HOTAIR sensitizes breast cancer cells to ionizing radiation through activating miR-218

**DOI:** 10.1042/BSR20181038

**Published:** 2019-04-05

**Authors:** Xuguang Hu, Dan Ding, Jiayi Zhang, Jianguo Cui

**Affiliations:** 1 Department of Gastrointestinal Surgery, Changhai Hospital, Shanghai, China; 2 Department of General Surgery, Changhai Hospital, Naval Medical University, Shanghai 200433, China; 3 School of Basic Medical Sciences, Navy Medical University, Shanghai 200433, China; 4 Department of Radiation Medicine, Faculty of Naval medicine, Naval Medical University, Shanghai 200433, China

**Keywords:** lncRNA HOTAIR, miR218, radiotherapy, radiosensitization

## Abstract

Radiotherapy is a major therapeutic strategy for breast cancer, while cancer radioresistance remains an obstacle for the successful control of the tumor. Novel radiosensitizing targets are to be developed to overcome radioresistance. Recently, long non-coding RNAs (lncRNAs) were proved to play critical roles in cancer progression. Among all, lncRNA HOTAIR was found to participate in cancer metastasis and chemoresistance. In the present study, we aimed to investigate the radiosensitizing effects of targeting HOTAIR and the underlying mechanism. Our data showed that HOTAIR (HOX antisense intergenic RNA) was up-regulated in breast cancer cells and tissues, and the expression of HOTAIR increased following irradiation. Knockdown of HOTAIR inhibited cell survival and increased cell apoptosis in response to ionizing radiation. Moreover, compared with control group, radiation induced more DNA damage and cell cycle arrest in HOTAIR knockdown cells. Finally, we found that the radiosentizing effects of HOTAIR were related to the up-regulation of miR-218, a ceRNA of HOTAIR. In conclusion, our finding showed that HOTAIR inhibition sensitizes breast cancer cells to ionizing radiation, induced severe DNA damage and activated apoptosis pathways, suggesting a possible role of HOTAIR as a novel target for breast cancer radiosensitization.

## Introduction

Breast cancer is the most frequent cancer among women worldwide [1]. For patients receiving breast-conserving surgery and node-positive patients, radiotherapy plays critical roles in the recurrence control and exhibits beneficial influence on the overall survival [1,2]. However, irradiation on normal tissues with high doses of radiation results in severe toxicity, which limits the further application of radiotherapy [2,3]. Novel strategies for radiosensitization in breast cancer were urgently required to reduce the radiation doses in radiotherapy.

Long non-coding RNAs (lncRNAs) (more than 200 nucleotides) are a class of newly identified non-coding transcripts, which were mistaken as transcriptional noise [4,5]. Recently, accumulating evidence suggests that lncRNAs play critical roles in regulating multiple biological processes through bridging the interaction and regulation among protein, DNA, as well as RNA expression [5]. And many lncRNAs were identified as oncogenic or tumor suppressors, which indicate critical roles of these non-coding transcripts. First, lncRNAs were demonstrated to regulate gene expressions as a cis-regulator, as it is correlated with its neighboring genes [6]. Further study has proved that lncRNA could bind to critical proteins through a direct interaction, and exert regulatory functions [7–10]. For example, lncRNA TDRG1 enhances tumorigenicity in endometrial carcinoma by directly binding with VEGF-A protein [11]. Recently, several studies showed that lncRNA can serve as a ‘sponge’ to titrate microRNAs and prevent them from binding to mRNAs [12]. And many lncRNAs were proved to bind to their ceRNA through base paired mechanism, and participate in various biological processes.

Out of cancer-associated lncRNAs, HOTAIR (HOX antisense intergenic RNA) is the most up-regulated in breast cancer [13]. And the aberrant expression of HOTAIR was related with the poor prognosis of breast [14,15]. HOTAIR could also regulate cancer proliferation as well as metastasis, and confer to tamoxifen resistance [16,17]. Through a ceRNA mechanism, HOTAIR inhibits miR-7 and regulates the EMT of breast cancer stem cells by down-regulating the STAT3 Pathway [18]. Previous study has also demonstrated that small peptides targeting HOTAIR inhibited growth of breast cancer cells [19]. It was reported that HOTAIR affected radiosensitivity in MDA231 cells [20], but the underlying mechanism remains unclear. In the present study, we found that down-regulation of HOTAIR sensitizes breast cancer cells to ionizing radiation through regulation on miR-218, which provided novel mechanism and target for breast cancer radiotherapy.

## Materials and methods

### Cells and transfection

Human breast cell lines MCF-7, SKBR3, and MDA-231 (ATCC, U.S.A.) were used in our study. MCF-7 and MDA231 cells were maintained in DMEM medium, and SKBR3 cells were maintained in RMPI1640 medium. All media were supplemented with 10% fetal bovine serum (Gibco, U.S.A.) supplemented with penicillin (100 U/ml) and streptomycin (100 μg/ml). Along the culture, cells were kept in a humid air at 37°C with the percentage of CO_2_ at 5%. In order to knock down HOTAIR, cells were transfected with HOTAIR shRNA (Genemediate Biological Tech., China) by using a Lipofectamine 3000 transfection reagent (Invivogen, U.S.A.) according to the manufacturer’s instructions.

### CCK8 assay

After different treatments, cell proliferation was measured by using a CCK8 kit (Beyotime, China). Six thousand cells were seeded in each well of 96-well plates, at 12 h after which cells were irradiated with different doses of radiation. Finally, cells were incubated with CCK8 solution and the density was measured with 570 nm light. Experiments were conducted for five independent times.

### Cell survival assay

Cell survival was determined with a colony formation assay. Briefly, cells were seeded at indicated number (according to the radiation doses) in 6-well plates, after which cells were irradiated with a ^60^Co irradiator. Ten days later, cells were fixed and stained with 0.1% crystal violet. And the colonies were counted manually.

### Cell apoptosis and cell cycle

For cell apoptosis analysis, an Annexin V-FITC and PI apoptosis kit (Beyotime, China) were used according to the manufacturer’s instructions. Briefly, after different treatments, cells were stained with Annexin V-FITC for 20 min at room temperature in dark. Just before sampling, cells were stained with PI and subjected to flowcytometry analysis (BD, U.S.A.). For cell cycle analysis, fixed cells were treated with RNase and PI for 30 min, and subjected to flowcytometry analysis as previously described [21].

### Tissue samples and real-time PCR assay

Paired breast cancer tissues and adjacent normal tissues resected surgically used for qRT-PCR were collected from 10 breast cancer patients during operation at Changhai Hospital (Shanghai, China). Then the samples were kept in −80°C, and were used for RNA extraction as well as real-time PCR analysis. For tissues and cells, after extraction of RNA with TRIZOL reagent (Invivogen, U.S.A.), cDNA was synthesized with a first strand cDNA synthesis kit (Takara, Dalian, China) according to the manufacturer’s instructions. And the expression of lncRNA HOTAIR was measured by using a SYBR real-time PCR kit (Takara, Dalian, China). The primers were used from previous study [22] listed as follows: GAPDH, F: GCACCGTCAAGGCTGAGAAC, R ATGGTGGTGAAGACGCCAGT; HOTAIR, F: GGTAGAAAAAGCAACCACGAAGC, R: ACATAAACCTCTGTCTGTGAGTGCC.

### Western blotting

Western blotting was used to detect the protein expression level as previously described [23]. In our present study, proteins in apoptosis pathway including caspase 3 (1:1000, CST, U.S.A.), bax (1:1000, CST, U.S.A.), and bcl2 (1:1000, CST, U.S.A.) were determined. And β-actin (1:2000, Abcam, U.S.A.) was used as an internal control.

### Statistical analysis

All the experiments were conducted at least for three independent times. And the data were expressed as mean ± SEM. The data were analyzed by two-way ANOVA followed by two-tailed Student’s *t*-test. *P*<0.05 was considered as statistical significant.

## Results

### Expression of lncRNA HOTAIR in breast cancer tissues and cell lines

**Figure 1:**
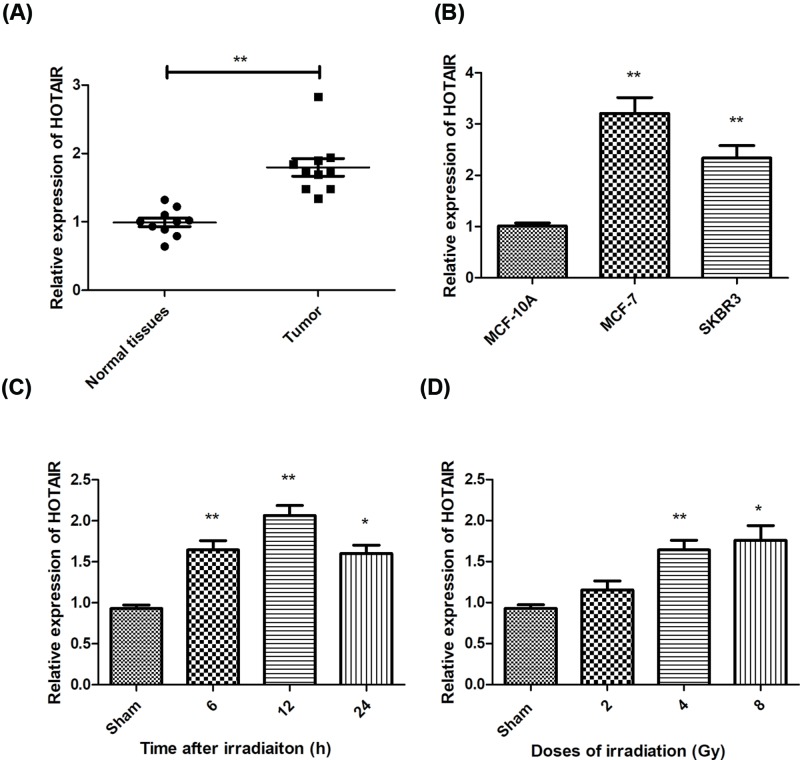
Expression of lncRNA HOTAIR in breast cancer tissues and cells Expression of lncRNA HOTAIR was examined by real-time PCR assay in breast cancer tissues and normal tissues (**A**), as well as in breast cancer cells (**B**). We also measured the level of HOTAIR at different times in MCF-7 cells in response to different doses of ionizing radiation (**C** and** D**). For kinetics of HOTAIR expression, cells were irradiated with a single radiation of 8Gy. *P<0.05, **P<0.01 vs the control groups.

lncRNA HOTAIR was found to be up-regulated in many tumors. First, we examined the expression of HOTAIR in 10 paired breast cancer tissues and found that HOTAIR increased significantly in cancer tissues, compare with the normal tissues (Figure 1A). Then we compared the level of HOTAIR in different breast cancer cell lines (Figure 1B). To study the response of HOTAIR to ionizing radiation, we checked the expression of HOTAIR in cells exposed to different doses of radiation. Our data showed that radiation elevated the expression of HOTAIR at 6, 12, and 24 h after irradiation, which peaked at 12 h (Figure 1C). For dose response, we found that other than 2, 4, and 8 Gy irradiation caused significant increase in HOTAIR (Figure 1D).

### Knockdown of lncRNA HOTAIR increased cellular sensitivity to IR

**Figure 2:**
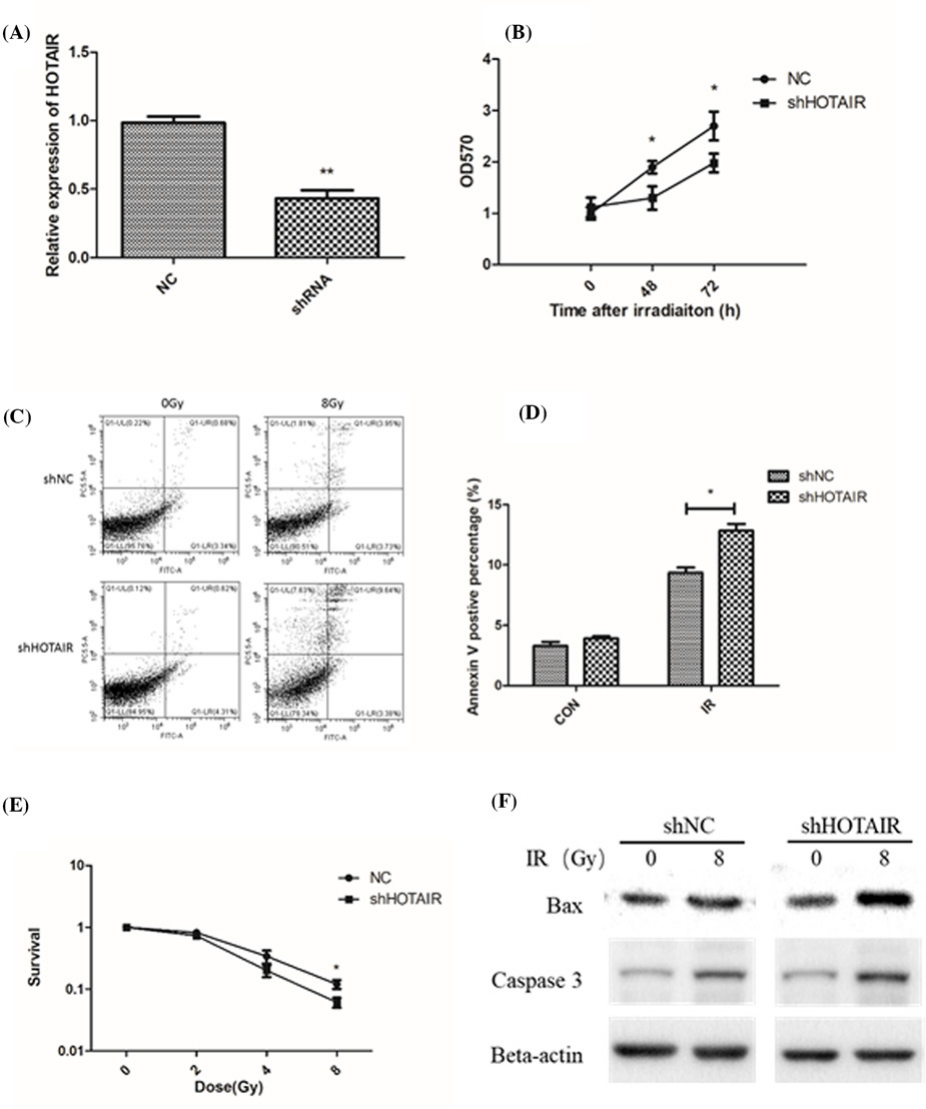
Knockdown of lncRNA HOTAIR increased cellular sensitivity to IR A siRNA was used to knock down HOTAIR in MCF7 cells (**A**). Cell proliferation was measured by using a CCK8 assay in wild-type and HOTAIR knockdown cells (**B**). We measured cell apoptosis in MCF7 cells and HOTAIR knockdown cells using an Annexin V and PI double staining kit (**C** and **D**). (**E**) Cell survival data in irradiated normal control cells and HOTAIR knockdown cells. (**F**) Representative images of Western blot analysis of bax and caspase 3 with/without radiation treatment in normal and HOTAIR knockdown cells. *P<0.05, **P<0.01 vs the control groups.

To study the role of HOTAIR in radiosensitivity, we used a siRNA to knock down the level of HOTAIR (Figure 2A). It was found that lncRNA HOTAIR knockdown resulted in slower proliferation and survival after exposure to radiation, compared with the normal control group (Figure 2B,C). HOTAIR down-regulation also increased radiation-induced cell apoptosis (Figure 2D), and activated apoptotic signaling proteins, such as bax and caspase 3 (Figure 2E). These data indicate that targeting HOTAIR might be a potential strategy for radiosensitization in breast cancer.

### Knockdown of lncRNA HOTAIR promoted DNA damage and cell cycle arrest

**Figure 3:**
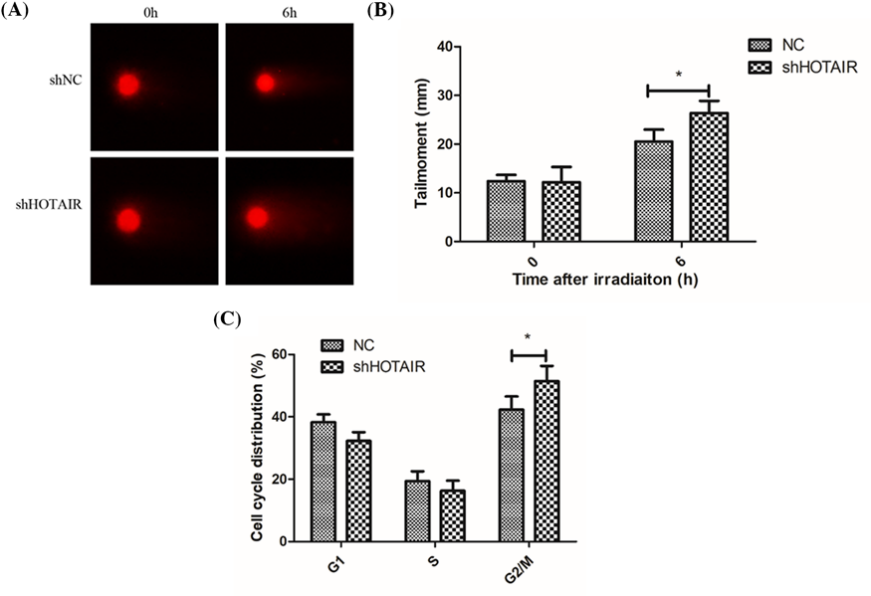
Knockdown of lncRNA HOTAIR promoted DNA damage and cell cycle arrest Comet assay showed that cells with HOTAIR knockdown were more susceptible to ionizing radiation (**A, B**). Cell cycle assay showed that more G2/M arrest was found in HOTAIR knockdown cells (**C**). *P<0.05 vs the control groups.

Ionizing radiation causes DNA damage, mainly including double-strand break and single-strand break, which initiates the downstream signaling pathways involving cell apoptosis and cell cycle arrest. We used a comet assay to determine DNA damage caused by irradiation, and found that the HOTAIR deficient cells were more susceptible to radiation-induced DNA damage (Figure 3A,B). Cell cycle assay showed that more G2/M arrest was induced in the HOTAIR knockdown cells (Figure 3C), which indicate accumulation of unrepaired DNA damage.

### lncRNA HOTAIR regulates cellular radiosensitivity through regulating miR-218

**Figure 4:**
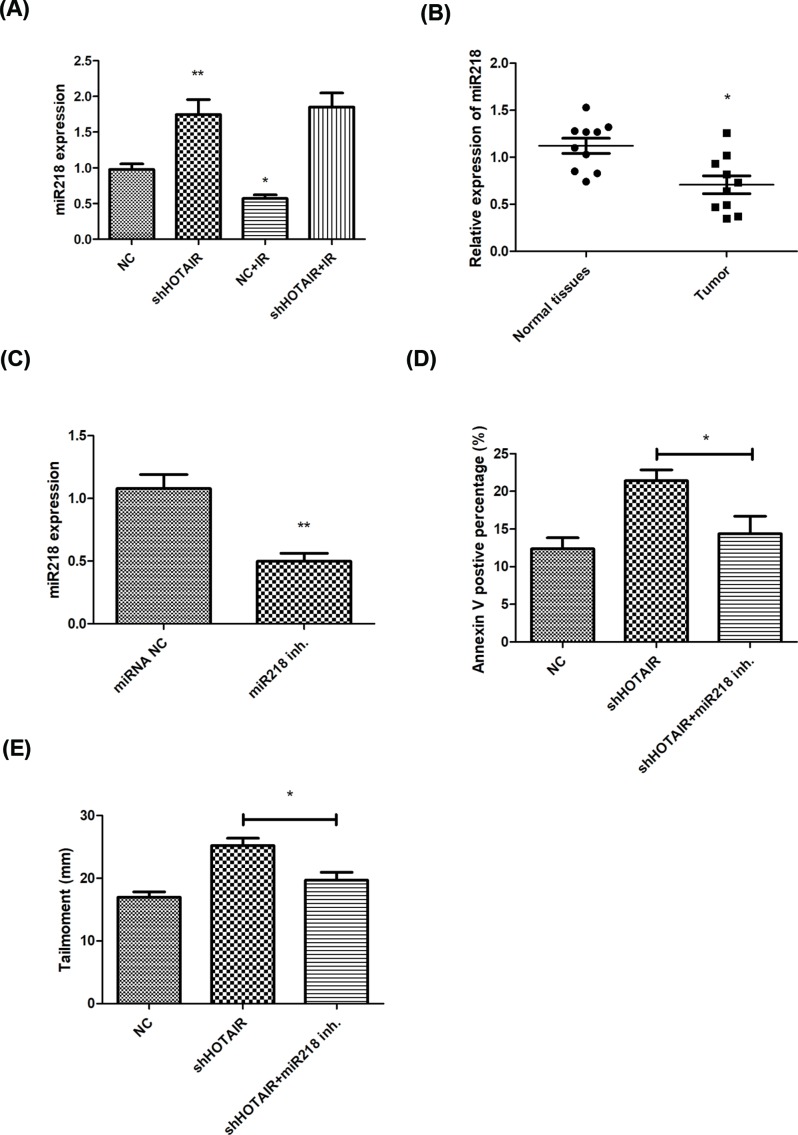
lncRNA HOTAIR regulates cellular radiosensitivity through interacting with miR-218 miR-218 was found increased in HOTAIR knockdown cells after 8 Gy irradiation, and decreased in breast cancer tissues (**A** and** B**). And miR-218 inhibitor attenuated radiation induced apoptosis and DNA damage in HOTAIR knockdown cells (**C**–**E**). *P<0.05, **P<0.01 vs the control groups.

lncRNA could work as a ceRNA sponging with miRNA through which participating in many biological processes. It has been reported that lncRNA HOTAIR suppressed miR-218 and contributes to 5FU resistance. In our present study, we found that miR-218 was up-regulated in HOTAIR knockdown cells and down-regulated in tumors (Figure 4A, B), and miR-218 level was significantly inhibited by its inhibitor (Figure 4C). miR-218 inhibitor reduced cell apoptosis in HOTAIR knockdown cells after irradiation (Figure 4D). miR-218 also promoted the repair of radiation-induced DNA damage in HOTAIR deficient groups (Figure 4E). These data indicated that lncRNA HOTAIR-miR-218 signaling might accounts for the mechanism of radiosensitization.

## Discussion

Overcoming radioresistance is critical for controlling tumor growth as well as lowering the radiation doses in normal tissues during radiotherapy. However, there is no ideal therapeutic strategy for radiosensitization in breast cancer in clinical. Recently, lncRNA was proved to exert significant roles in the development and progression of cancer. Among all lncRNAs, HOTAIR was up-regulated in many cancers, including gastrointestinal stromal tumors, cervical cancer, lung cancer, as well as breast cancer [24–27]. In this study, we demonstrated that lncRNA HOTAIR plays a critical role in radiation resistance of breast cancer cells. And knockdown of HOTAIR increased cellular sensitivity to ionizing radiation through miR-218.

Recently, several studies have demonstrated that lncRNA HOTAIR was involved in the regulation of breast cancer proliferation, metastasis, and invasion. Xue et al. reported that lncRNA HOTAIR activates ER signaling and confers tamoxifen resistance in breast cancer [28]. It was also demonstrated that HOTAIR single nucleotide polymorphism was a marker of the risk of breast cancer [29]. And several studies have identified that inhibition of HOTAIR by tumor-specific peptides or calycosin and genistein exhibited tumor therapeutic effects on breast cancer [,^30^]. Several miRNAs were also reported to regulate growth of breast cancer cells through inhibiting HOTAIR [18]. However, there is no study showing the effects of HOTAIR on radiosensitivity of breast cancer cells. In the present study, we found that lncRNA was up-regulated in breast cancer cells, and knockdown of HOTAIR increased cell apoptosis induced by IR. HOTAIR down-regulation also results in more DNA damage compared with the single radiation groups. It was also found that HOTAIR confers to platinum resistance through activation of DNA damage response, HOTAIR also activates NF-kB [31]. DNA damage induced the expression of HOTAIR was also dependent on the status of p53 [32], which indicates that HOTAIR plays a role in p53-regulated DNA damage response. HOTAIR was found to link DNA damage and NF-kB signaling [31], which also promote cell survival in response to irradiation. However, the specific mechanism of HOTAIR in DNA damage and radioresistance remains unclear.

Mechanically, lncRNA could work as a ceRNA sponging with its target miRNA and exert different functions. It has been demonstrated that lncRNA HOTAIR binds with many miRNA including miR-130a, miR-218, miR-7, miR-203, etc. in gallbladder cancer, hepatocellular carcinoma, or renal cancer cells [33–35]. Binding sites of these miRNAs have been identified within the sequence of HOTAIR. And after binding, HOTAIR reduced the level of miRNA through a ceRNA sponging mechanism, which results in a up-regulation of miRNA targets and changes in signaling transduction. In our study, we found that miR-218 might be a potential target of lncRNA HOTAIR. Moreover, miR-218 was found to inhibit tumor suppressing genes and regulate chemoresistance in breast cancer cells [36,37]. Surprisingly, miR-218 was also reported to increase radiosensitivity in human cervical cancer [38]. Thus in this study, we investigated the role of HOTAIR–miR-218 axis on radiosensitivity of breast cancer cells. And we found that miR-218 was up-regulated in HOTAIR knockdown cells, and the up-regulation of miR-218 promoted cells apoptosis and DNA damage after irradiation, suggesting miR-218 as a potential target of HOTAIR in the regulation of radiosensitivity of breast cancer. It has been proved that miR-218 is tumor suppressive, and inhibits gastric cancer as well as oral cancer [39,40]. miR-218 also regulates DNA damage and cisplatin resistance through targeting BRCA1, which is involved in DNA repair [41]. Moreover, miR-218 inhibits carboplatin resistance in A549 and H1975 cells through targeting Mcl-1 and Survivin, suggesting a role of miR-218 in apoptosis [42]. These findings suggest that HOTAIR–miR-218 interaction might account for the mechanism of radiosensitivity and DNA damage repair.

In conclusion, our data showed that lncRNA HOTAIR was up-regulated in breast cancer, and HOTAIR inhibition resulted in a radiosensitive phenotype. Knockdown of HOTAIR caused more cell apoptosis and DNA damage through releasing its sponging miR-218. Our data suggest a novel therapeutic target of lncRNA HOTAIR-miR-218 axis in breast cancer.

## Abbreviations

HOTAIR: HOX antisense intergenic RNA
lncRNA: long non-coding RNA
